# Chronotype and Sleep Quality in College Students Undergoing Clinical Placement: A Moderated Moderation Model of Sleep Reactivity and Resilience

**DOI:** 10.1097/jnr.0000000000000681

**Published:** 2025-06-23

**Authors:** Renny Wulan Apriliyasari, Jia-Wei Liu, Chia-Wen Chou, Jin-Hua Chen, Pei-Shan Tsai

**Affiliations:** 1 School of Nursing, College of Nursing, Taipei Medical University, Taipei, Taiwan; 2 Department of Nursing, Institute of Health Technology, Cendekia Utama Kudus, Kudus, Indonesia; 3 Graduate Institute of Data Science, College of Management; 4 Institutional Research Center, Office of Data Science; 5 Pulmonary Research Center, Wan Fang Hospital, Taipei Medical University, Taipei, Taiwan; 6 Department of Nursing and Research Center in Nursing Clinical Practice, Wan Fang Hospital, Taipei Medical University, Taipei, Taiwan; 7 Research Center of Sleep Medicine, Taipei Medical University Hospital, Taipei, Taiwan; † Contributed equally

**Keywords:** chronotype, sleep quality, sleep reactivity, resilience, college student

## Abstract

**Background:**

Chronotype, also referred to as morningness and eveningness, describes the natural preference of the body for wakefulness or sleep at different times during a 24-hour period. Individuals demonstrating late chronotypes and low resilience tend to have poor sleep quality, and the association between late chronotypes and sleep quality is known to be moderated by sleep reactivity. The mediating roles of sleep reactivity and psychological resilience in the association between chronotype and sleep quality in college students under situations of high stress have yet to be investigated.

**Purpose:**

This study was designed to evaluate the degree to which resilience moderates the moderating role of sleep reactivity on the association between chronotype and sleep quality in college students undergoing clinical placement.

**Methods:**

A cross-sectional study involving 225 college students undergoing clinical placement was conducted. The Chinese version of the Pittsburgh Sleep Quality Index was used to assess sleep quality, chronotype was assessed using the Morningness–Eveningness Questionnaire, sleep reactivity and resilience were respectively assessed using Ford’s Insomnia Response to Stress Test and Brief Resilience Scale, and the SPSS PROCESS macro Version 4.3 (Hayes) was employed in moderated moderation analysis.

**Results:**

Resilience was shown to moderate the relationship between sleep reactivity and sleep quality (β=0.079, *p*=.039) as well as the moderating role of sleep reactivity in the chronotype–sleep quality relationship (β=−0.002, *p*=.027).

**Conclusions:**

In this study, sleep reactivity and chronotype both exhibited inverse effects on sleep quality in the moderate- and high-resilience groups. However, those in the low-resilience group with high sleep reactivity exhibited low sleep quality regardless of chronotype. Considering these findings, sleep reactivity and resilience should be adequately monitored during interventions designed to enhance sleep quality.

## Introduction

Adequate sleep is crucial to maintaining cognitive capabilities, emotional stability, and general health (Scott et al., 2021). Across the various stages of life, a significant proportion of the population suffers from suboptimal sleep quality, with reported prevalence rates fluctuating between 19% and 47.12% (P. Chen et al., 2024; Du et al., 2023; Phiri et al., 2023). Alarmingly, evidence points to a particularly worrying pattern of poor sleep quality among college students (Binjabr et al., 2023). The college years represent a critical phase of development that bridges late adolescence and early adulthood. In college students, a combination of inadequate sleep practices and the pressures of academic life may detrimentally impact on sleep quality (Y. Wang et al., 2023).

Health science students, particularly medical and nursing students, tend to report insufficient sleep duration, excessive daytime sleepiness, and low sleep quality (Correia et al., 2022). College students undergoing clinical placement are more likely to suffer from poor sleep quality than either their peers who have yet to undergo this process (Khero et al., 2019) or non-health-science students (Nadeem et al., 2018). Furthermore, insufficient sleep may have long-term adverse effects on the academic performance, years of study, mental health, and physical health of students (Paudel et al., 2022).

Chronotype, also referred to as morningness and eveningness, is a human trait representing an individual’s circadian phases that emerge when the individual’s biological functions, hormone levels, body temperature, cognitive abilities, and eating and sleeping patterns are active (Levandovski et al., 2013). The concept of chronobiology highlights that the primary circadian clock resides in the suprachiasmatic nucleus within the anterior hypothalamus. This clock governs numerous circadian rhythms, including the sleep–wake cycle (Montaruli et al., 2021). Human sleep–wake cycles are regulated by sleep homeostasis and circadian processes. Discrepancies between an individual’s internal circadian rhythm and their external daily schedule can lead to sleep disturbance (Dijk & Lockley, 2002). Individuals with a morning preference (aka morning-type) often wake up early and experience peak mental and physical performance during the morning hours. Conversely, evening-type (aka late chronotype) individuals, also known as “owls,” typically wake up later and reach their peak performance toward the end of the day, often in the late evening (Adan et al., 2012). Several studies have examined the effects of chronotype on health, revealing that evening-type individuals have lower sleep quality than morning-type individuals across different population groups, including general (Salfi et al., 2022) and college student (Zhu et al., 2023) populations.

The results of a literature review study indicate mental health (i.e., perceived stress) is a contributing factor of sleep quality among college students (F. Wang & Bíró, 2021). Sleep reactivity, defined as stressful situations that impede falling asleep and staying asleep (Kalmbach, Cuamatzi-Castelan, et al., 2018), has been shown to moderate the relationship between the evening chronotype and low sleep quality in healthy daytime workers, individuals with mood disorders, and other population subgroups (C. H. Chen et al., 2022; Saalwirth & Leipold, 2021). The sleep homeostasis model proposed by Borbély, 1982 is predicated on the presumption that variations in individual sleep reactivity impact homeostatic control of sleep, with those with heightened sleep reactivity at higher risk of disrupted homeostatic regulation, which hinders their ability to achieve restful sleep and sustain sleep continuity. In this model, people with evening chronotypes and elevated sleep reactivity are particularly prone to delays in falling asleep and interruptions in sleep continuity (Fitzpatrick et al., 2023), attributable to both circadian rhythm misalignment and compromised sleep homeostasis (Borbély, 2022). In college students, sleep reactivity is particularly prevalent among those undergoing clinical placement. This group faces numerous stressors and challenges such as academic demands, exposure to patients and illnesses, frequent exams, and extensive curricula, all of which exacerbate sleep disturbance (Safhi et al., 2020). Furthermore, sleep reactivity may interrupt the body’s natural circadian rhythm, resulting in low sleep quality and negatively affecting academic performance (Phillips et al., 2017).

Clinical psychology has increasingly focused in recent years on psychological resilience, which is regarded to be critical for adaptively overcoming stress while maintaining normal mental and physical functions. The stress-buffering model introduced by Cohen and Wills, 1985 highlights how social support and coping resources can lessen the effects of stress on sleep. This framework suggests that individuals with higher resilience levels are better equipped to manage sleep disturbances and challenges, underscoring the need to account for personal differences in coping mechanisms and social networks. According to Ellison et al. (2019), these individual variations in coping resources and social support are crucial to consider when investigating the link between stress and sleep quality. Supporting this, Liu et al. (2016) found a relationship between resilience and sleep issues, indicating individuals with lower resilience tend to experience more significant sleep disturbances and shorter time to fall asleep. Individuals with low levels of sleep quality tend to experience more severe resilience-related problems, including greater difficulties with goal-oriented planning, time and routine organization, and clear goal planning and formulation, than their peers with high levels of sleep quality (Palagini et al., 2018). In term of chronotype conditions, a previous study showed morningness, after adjusting for sleep quality and other confounding variables, to correlate significantly with resilience in college students (S. J. Lee et al., 2016). Another study revealed psychological resilience to moderate the relationship between chronotype and mental condition (Zhou et al., 2021). Based on these prior findings, the role of resilience in the relationship between chronotype and sleep quality deserves further investigation.

Despite substantial evidence indicating a significant association between chronotype and sleep quality, several studies have reported conflicting results (C. Y. Lee et al., 2015; Salfi et al., 2022). This discrepancy may be attributed to the aforementioned inadequate investigation to date of the effect of sleep reactivity on resilience. Moreover, studies examining the interactions among chronotype, sleep reactivity, and resilience in terms of their effects on sleep quality in college students are limited. Rather, studies to date have largely focused on the interaction between stressful life conditions and resilience and the effect of these variables on sleep quality in healthy adolescents (Y. Li et al., 2019) and adults (Liu et al., 2016) as well as the interaction between chronotype and resilience in the context of psychological health (Zhou et al., 2021). By elucidating the correlations between chronotype, sleep reactivity, and resilience, respectively, and sleep quality, the authors aim to deepen scholarly insight into the psychological facets of sleep health in college students, with a particular focus on those engaged in clinical placements. This exploration is crucial for designing targeted interventions that not only foster healthier sleep habits but also significantly enhance well-being in students. Through this research, we seek to contribute to a better understanding of sleep dynamics in students to guide the development of strategies to improve their academic performance, health, and overall quality of life.

Therefore, in this study, a moderated moderation model was proposed and tested. Specifically, the moderating effect of resilience on the moderating role of sleep reactivity on the association between chronotype and sleep quality was investigated in college students undergoing clinical placement.

## Methods

### Participants and Procedure

A cross-sectional study involving 225 participants was conducted to examine the role of sleep reactivity and resilience in the relationship between chronotype and sleep quality in college students undergoing clinical placement in northern Taiwan. College students undergoing clinical placement who agreed to participate were recruited. The inclusion criteria were as follows: 20–30 years of age, undergoing clinical placement currently or within the last 2 weeks, and understanding and being able to use Mandarin Chinese in speaking and writing. The exclusion criteria were as follows: working night shifts, being pregnancy, having a severe disease (e.g., stroke, cancer), having a severe mental illness other than depression, or undergoing light therapy.

An online survey was conducted between September 1 and October 10, 2022. After being informed of the research context, purpose, and eligibility criteria, the participants provided informed consent by ticking off the following question: “I understand the information and agree to participate in the study.” Subsequently, they answered questions pertaining to basic demographics data and completed the questionnaires. Those participants who missed any questions were regarded as nonrespondents, and their data were excluded from analysis.

### Sample Size and Sampling Technique

A convenience sampling method was used, and the rule-of-thumb principle was applied to estimate the requisite sample size. A total of three independent variables (chronotype, sleep reactivity, and resilience), three pairwise interaction terms (chronotype and sleep reactivity, sleep reactivity and resilience, and resilience and chronotype), one 3-variable interaction term (chronotype, sleep reactivity, and resilience), and 11 confounders (age, gender, department of study, marital status, employment status, smoking history, alcohol consumption, sleep medication history, exercise habit, co-sleeping partner, and social jetlag) were included in this study, resulting in 18 variables. Therefore, the minimum sample size determined to be 180. To account for potential nonrespondents and a dropout rate of 20%, 216 students meeting the inclusion criteria were recruited and enrolled as participants.

#### Chronotype

Chronotype was evaluated in this study using the Chinese version of the Morningness–Eveningness Questionnaire (MEQ). The MEQ is a self-administered questionnaire containing 19 questions on preferred personal bedtime, wake-up time, and activity time. Answers to each question are scored on a Likert scale with endpoints ranging from 16 to 86 to capture differences in individual circadian rhythms (S.-X. Li et al., 2011), with higher scores representing a stronger tendency toward morningness. The Chinese version of MEQ has a satisfactory internal consistency, with a Cronbach’s alpha of .769 in S.-X. Li et al. (2011) and .76 in this study.

#### Sleep Reactivity

Sleep reactivity was evaluated using the Ford Insomnia Response to Stress Test (FIRST; Drake et al., 2004), which is designed to measure the degree to which sleep is affected by stress in individuals experiencing stressful conditions. The FIRST is a self-administered questionnaire with a total of nine questions. Answers to each are scored on a 4-point scale, with endpoints of 1 (*unlikely to happen*) to 4 (*very likely to happen*). The total score ranges from 9 to 36, with higher scores indicating a greater impact of stress on sleep disruption. The authors had obtained permission from the original author to translate the FIRST into a Chinese version and use the Chinese version in research. This version showed excellent internal consistency (Cronbach’s alpha of .81) in this study.

#### Resilience

Resilience was evaluated using the Chinese version of the six-item Brief Resilience Scale (Fung, 2020). Each item is scored on a 5-point Likert scale, with endpoints of 1 (*strongly disagree*) to 5 (*strongly agree*). Items 1, 3, and 5 are forward questions, while Items 2, 4, and 6 are reverse questions. Reverse questions are scored in reverse, resulting in a total score ranging from 5 to 25, with higher scores indicating higher individual resilience, which reflects the respondent’s ability to adapt and recover from adverse events. This scale was previously found to have satisfactory construct validity (Keiser–Meyer–Olkin score of .75) and internal consistency (Cronbach’s alpha of .71; Fung, 2020) and was found to have excellent internal consistency (Cronbach’s alpha of .87) in this study.

#### Sleep Quality

Sleep quality was evaluated using the Chinese version of the Pittsburgh Sleep Quality Index (CPSQI; Tsai et al., 2005). The CPSQI comprises 19 items covering the seven components of subjective sleep quality, sleep latency, sleep duration, habitual sleep efficiency, sleep disturbance, use of sleeping medication, and daytime dysfunction. Each item is scored between 0 and 3, with a score of 3 indicating severe difficulty (Buysse et al., 1989). The global CPSQI score (ranging from 0 to 21) is the sum of the seven component scores, with higher global scores indicating lower subjective sleep quality. The CPSQI has previously demonstrated satisfactory test–retest reliability over a 14- to 21-day period, with a coefficient of .85 for all individuals and .77 for the primary insomnia population (Tsai et al., 2005). A cutoff point score of>5 is used to indicate poor sleep quality (Tsai et al., 2005).

In addition, the recommendations of the National Sleep Foundation were used to determine the cutoff values for age-appropriate sleep duration, sleep latency, and sleep efficiency (Hirshkowitz et al., 2015; Ohayon et al., 2017). For young adults, 7–9 hours of sleep per day, sleep latency of no more than 30 minutes, and a habitual sleep efficiency of ≥ 85% are recommended (Hirshkowitz et al., 2015; Ohayon et al., 2017).

### Confounders

The following sociodemographic characteristics were collected and treated as potential confounders: age, gender, department of study (medicine and nursing department vs. other departments), marital status (married vs. unmarried), employment status (students vs. part-time job), smoking history (nonsmoker vs. current smoker), alcohol consumption (yes vs. no), sleep medication history (yes vs. no), exercise habit (yes vs. no), co-sleeping partner (yes vs. no), and social jetlag. Smoking history was originally based on the answer “never,” “former smoker,” and “current smoker” and then categorized into “nonsmoker” and “current smoker” as there were no former smokers in the study sample. Alcohol consumption was classified into “yes” if participants had consumed alcohol during the last month and “no” if they had never consumed alcohol. The same categorization was also applied to sleep medication history, exercise habits, and co-sleeping partners. Social jetlag was determined by calculating the difference in the midpoint of sleep between weekdays and weekends (Wittmann et al., 2006). In this study, a cutoff value of 2 hours was used to categorize social jetlag (Hena & Garmy, 2020), distinguishing social jetlag into a binary choice between “<2 hours” and “≥ 2 hours.”

### Statistical Analysis

All statistical analyses in this study were conducted using IBM SPSS Statistics 28.0 (IBM Corp., Armonk, NY, USA). Data for continuous variables were presented as mean and *SD*, while data for categorical variables were presented in terms of numbers and frequencies. The association of each confounder with sleep quality was analyzed using an independent *t* test, while associations between variables, including chronotype, sleep reactivity, resilience, and sleep quality, were analyzed using Pearson’s correlation. Multivariate linear regression analysis was conducted to examine the relationship between primary variables, with adjustment for confounders (confounders with *p*<.25 in the bivariate analysis were included). Hierarchical and stepwise linear regression analyses were used to identify the effect of the confounders in each model. In addition, the PROCESS macro Version 4.3 (Hayes) was used to examine the moderated moderation model of the effects of sleep reactivity and resilience in the relationship between chronotype and sleep quality. Statistical significance was set at *p*<.05.

### Ethical Approval

The protocol for this study was approved by the Joint Institutional Review Board of Taipei Medical University (No. TMU-JIRB N202206100).

## Results

### Sociodemographic Characteristics

Of the 225 participants enrolled, most were women (76%), and the average age was 22.02 (*SD*=1.61) years. Half (109; 48.4%) were studying in medicine or nursing departments, and 116 (51.6%) were studying in other departments. In terms of health behavior, most were nonsmokers (96.9%), consumed alcohol (56.4%), used sleep medication (43.1%), exercised at least once or twice every month (77.5%), and had less than 2 hours of social jetlag (63.6%).

The majority of participants reported having fairly good sleep quality (58.2%), sleep latency of 30 minutes or less (87.6%), and a habitual sleep efficiency of ≥ 85% (78.2%). However, only 43.6% met the recommendation for appropriate sleep duration, 20% reported experiencing sleep disturbance at least one to two times per week, and 24.9% reported experiencing daytime dysfunction at least one to two times per week. Furthermore, a majority of the participants (*n*=125, 56%) earned a CPSQI score of>5, indicating poor sleep quality, with an average score of 5.95 (*SD*=2.54) and participant CPSQI total scores ranging between 0 and 14. Sleep quality (i.e., global CPSQI score) by sociodemographic characteristics is presented in Table 1.

**Table 1:** Sleep Quality (i.e., Global CPSQI Score) by Sociodemographic Characteristics (*N*=225)

Variable	*n* (%)	Mean (*SD*)	*r* or *t*	*p*
		Sleep Quality		
Age (years, *M* and *SD*)	22.02 (1.61)	—	*r*=.012	.012
Gender			−1.052	.294
Male	54 (24.0)	5.63 (2.23)		
Female	171 (76.0)	6.05 (2.63)		
Department of study			−0.284	.777
Medicine and nursing department	109 (48.4)	5.90 (2.55)		
Others departments	116 (51.6)	6.00 (2.53)		
Marital status			−0.810	.419
Unmarried	224 (99.6)	5.93 (2.54)		
Married	1 (0.4)	8.00 (-)		
Employment status			0.548	.584
Student	186 (82.7)	5.99 (2.50)		
Part time	39 (17.3)	5.74 (2.74)		
Smoking history			−1.488	.186
Non smoker	218 (96.9)	5.87 (2.44)		
Current smoker	7 (3.1)	8.29 (4.27)		
Alcohol consumption			−1.284	.201
Yes	127 (56.4)	6.14 (2.33)		
No	98 (43.6)	5.69 (2.78)		
Sleep medication history			−0.626	.532
Yes	97 (43.1)	5.82 (2.43)		
No	128 (56.9)	6.04 (2.63)		
Exercise habit			1.364	.174
Yes	174 (77.5)	5.82 (2.48)		
No	51 (22.5)	6.37 (2.71)		
Co-sleeping partner			0.684	.495
Yes	44 (19.6)	6.18 (2.47)		
No	181 (80.4)	5.89 (2.56)		
Social jetlag			−2.447	.015
<2 hours	143 (63.6)	5.64 (2.38)		
≥ 2 hours	82 (36.4)	6.49 (2.72)		
Chronotype (*M* and *SD*)	45.9 (7.8)	—	*r*=−.207	<.001
Sleep reactivity (*M* and *SD*)	21.0 (4.9)	—	*r*=.475	<.001
Resilience (*M* and *SD*)	18.7 (4.5)	—	*r*=−.380	<.001

### Preliminary Analysis

The means and *SD* of each variable and the correlation coefficients between variables are also given in Table 1. Pearson’s correlation analysis revealed the global CPSQI score to correlate significantly with chronotype, resilience, and sleep reactivity (*p*<.001). The results also revealed sleep reactivity and resilience to be significantly correlated (*p*<.001).

Multivariate hierarchical linear regression analysis was conducted with age and social jetlag treated as confounders. However, the results remained constant. As indicated by Model 3 (Table 2), when controlling for age and social jetlag, the global CPSQI score for the participants was negatively correlated with chronotype and resilience and positively correlated with sleep reactivity (all *p*<.05). When resilience (Model 2) and chronotype (Model 3) were added, the value of Δ*R* ^2^ increased, additionally explaining 25.5% and 27.3%, respectively, of the variance in sleep quality.

**Table 2:** Hierarchical Multiple Regression Analysis of Chronotype, Sleep Reactivity, Resilience, and Sleep Quality (*N*=225)

Variable	Sleep Quality								
	Model 1			Model 2			Model 3		
	β	*SE*	*p*	β	*SE*	*p*	β	*SE*	*p*
Constant	0.783	0.658	.235	4.049	1.181	.001	5.768	1.353	<.001
Chronotype	—	—	—	—	—	—	−0.048	0.019	.013
Sleep reactivity	0.245	0.030	<.001	0.197	0.033	<.001	0.201	0.033	<.001
Resilience				−0.121	0.037	.001	−0.100	0.037	.007
Δ*R* ^2^	.222			.255			.273		
*F*	65.026**			39.386**			28.990**		

*Note.* All models are controlled for age and social jetlag. Model 1=sleep reactivity; Model 2=sleep reactivity and resilience; Model 3=sleep reactivity, resilience, and chronotype; *SE*=standard error.

**p < .001.

### Moderated Moderation Analyses

**Figure 1:**
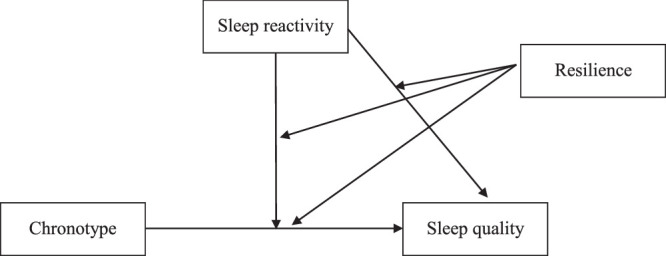
Hypothesized Moderating Effect of Sleep Reactivity and Resilience on the Relationship Between Chronotype and Sleep Quality

**Figure 2:**
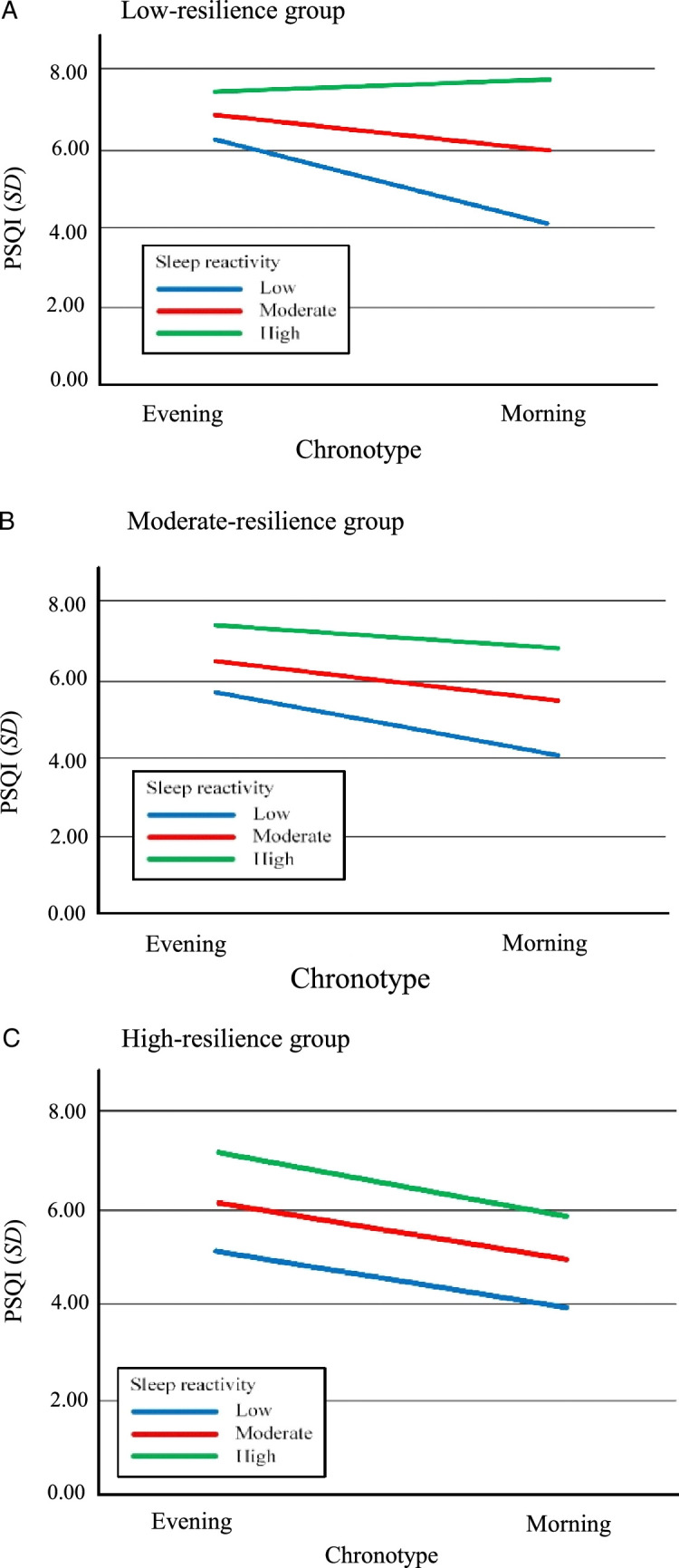
Relationships Between Chronotype, Sleep Reactivity, and Resilience and Sleep Quality *Note*. These figures illustrate the relationships between chronotype, sleep reactivity, and resilience, respectively, and sleep quality in the (A) low-resilience group, (B) moderate-resilience group, and (C) high- resilience group. PSQI = Pittsburgh Sleep Quality Index.

As shown in Figure 1, the PROCESS macro of SPSS Version 4.3 (Model 3) was used to evaluate a hypothesized moderated moderation (three-way interaction) model, with the results of the moderated moderation analysis shown in Table 3. Both sleep reactivity and resilience were found to moderate the relationship between chronotype and sleep quality (β=0.041 and β=0.362, respectively, *p*<.05). Moreover, resilience was found to moderate the relationship between sleep reactivity and sleep quality (β=0.079) as well as the moderating role of sleep reactivity on the relationship between chronotype and sleep quality (β=−0.002), with the moderated moderating model explaining 31.6% of the variance in the sleep quality. As shown in Figure 2, simple slope analysis revealed higher sleep reactivity and later chronotype to be associated with lower sleep quality in the high-resilience (1 *SD* above the mean) and moderate-resilience (*SD* within the mean) groups. However, in the low-resilience group (1 *SD* below the mean), those with high sleep reactivity had lower sleep quality than those with low and moderate sleep reactivity, irrespective of chronotype.

**Table 3:** Respective Moderating Role of Sleep Reactivity and Resilience in the Relationship Between Chronotype and Sleep Quality

Variable	Sleep Quality		
	β	*SE*	*p*
Constant	41.812	15.709	.008
Chronotype	−0.882	0.349	.012
Sleep reactivity	−1.552	0.687	.025
Resilience	−1.663	0.818	.043
Chronotype × Sleep Reactivity	0.041	0.015	.008
Chronotype × Resilience	0.362	0.018	.043
Sleep Reactivity × Resilience	0.079	0.038	.039
Chronotype × Sleep Reactivity × Resilience	−0.002	0.000	.027
*R* ^2^	.316	4.556	<.001

*Note.* Controlling for age and social jetlag.

## Discussion

The relationships between chronotype, sleep reactivity, and resilience, respectively, and sleep quality were investigated in this study. Sleep reactivity and resilience was found to exert a moderating effect on the relationship between chronotype and sleep quality in the sample (i.e., college students undergoing clinical placement). The findings also support the moderated moderation model of the effects of sleep reactivity and resilience on the relationship between chronotype and sleep quality. Also, resilience was found to moderate not only the relationship between sleep reactivity and sleep quality but also the moderating role of sleep reactivity on the relationship between chronotype and sleep quality.

The influence of chronotype on sleep quality, bedtime procrastination, and sleep hygiene awareness has been demonstrated in prior studies (e.g., Zhu et al., 2023). Also, students with evening chronotypes have been shown to exhibit relatively lower performance in morning classes (Jankowski et al., 2023). Overall, the findings in this study are consistent with those of previous studies that have found a strong correlation between chronotype and sleep quality in college students undergoing clinical placement. Consistent with the findings of prior studies that stress-related high sleep reactivity is strongly associated with low sleep quality (Almojali et al., 2017; Safhi et al., 2020), the findings of this study support a negative effect of sleep reactivity on sleep quality in college students undergoing clinical placement. The relationship between sleep reactivity and sleep quality may be attributed to activation of the hypothalamus–pituitary–adrenal axis, which is related to sleep and stress (Koren, 2021). Individuals with high sleep stress responses are susceptible to disruption of the cortical network in the brain because of higher cortisol levels, with 60% of these individuals experiencing symptoms of insomnia. Moreover, the sleep stress response mechanism may reduce the adaptive response to stress, resulting in the development of chronic insomnia (Kalmbach et al., 2018). Therefore, because clinical placement is considered a stressful event or experience, effective measures should be implemented to eliminate or even reduce the effect of clinical placement on sleep quality in college students with the late chronotype.

The findings of this study further support that psychological resilience plays an important role in the relationship of both chronotype and sleep reactivity to sleep quality in college students undergoing clinical placements. College students with lower sleep reactivity, the morning chronotype, and a moderate or high level of adaptive stress coping may thus be more likely to have a high quality of sleep, while those with low resilience and high sleep reactivity may be more likely to have low sleep quality irrespective of chronotype status. Sleep reactivity and resilience contribute to sleep quality in not only students (Sun et al., 2019) but shift nurses (Choi & Kim, 2022) as well. Restorative sleep promotes emotional adaptation, which is the nightly change in the brain’s limbic circuit (van der Helm et al., 2011), thus alleviating the burden of emotional memories by reducing their intensity and enhancing their manageability (Kindt & Soeter, 2018). By contrast, disturbed sleep inhibits these adaptive processes. Adverse effects are perceived for extended durations, with negative effects also exerted on brain activity for decades (Wassing et al., 2019). This condition may accelerate the onset of psychological disorders such as depression (Drake et al., 2014).

As discussed earlier in the text, sleep quality must be emphasized among college students, particularly those experiencing particularly stressful conditions (e.g., clinical placement) and who have the evening chronotype. By providing detailed information on various sleep quality domains, this study offers a clearer picture of participant sleep experiences. For instance, the high sleep efficiency reported by the majority of the participants provides insight that helps better elucidate the buffering effect of resilience on the negative effects of high sleep reactivity on sleep quality. In addition, the observed variability in sleep duration, the frequency of sleep disturbances, and the overall sleep quality score points to individual differences that may interact with chronotype and resilience to influence sleep outcomes. A meta-analysis study revealed that educational interventions, including cognitive behavioral therapy for insomnia, boosted quality of sleep among college students (Saruhanjan et al., 2021). Overall, the findings of this study support the incorporation of resilience and stress management strategies to mitigate sleep reactivity into interventions aimed at improving sleep quality. According to prior studies, self-guided stress management interventions should be provided to students to promote positive sleep-related outcomes (Amanvermez et al., 2022).

A strength of this study was that the moderated moderation analysis used facilitated the comprehensive examination of the complex relationships between chronotype, sleep reactivity, and resilience, respectively, and sleep quality in the study population. However, several limitations of this study must be acknowledged and considered. First, students who were working night shifts were excluded because their sleep results may have confounded the investigation into the role of chronotype. Future studies may extend the scope of investigation to elucidate the relationship between chronotype and sleep quality in the context of different work shifts. Second, the self-reported measures used to assess sleep quality may have been affected by response bias. Although all of the sleep-related instruments used in this study are reliable and commonly used, social desirability may still influence the answers given by participants. In addition, while data on subjective sleep experiences were not available, the utilization of sleep quality domains provided valuable insights into perceived sleep patterns and sleep disturbance. Third, the convenience sampling approach used in this study mean that the results may not be fully generalizable due to the oversampling of certain characteristics such as gender or department of study. Fourth, data were collected at a single time point using a cross-sectional study design. This may have limited the accuracy of conclusions related to causal relationships. Thus, future researchers should identify interventions that improve resilience and reduce sleep reactivity caused by stress. Fifth, sleep quality may be affected by other psychological factors such as anxiety and depression, which thus may mediate the relationship between stressful events and sleep quality. Future studies should develop multiple mediator models involving depression and anxiety symptoms.

### Conclusions and Implications

Sleep quality is strongly correlated with chronotype, sleep reactivity, and resilience, while sleep reactivity and resilience moderate the relationship between chronotype and sleep quality. Resilience was found to moderate not only the relationship between sleep reactivity and sleep quality but also the moderating role of sleep reactivity in the relationship between chronotype and sleep quality. These findings indicate the importance of educators and clinicians being cognizant and mindful that students with the night owl chronotype and those whose sleep is highly sensitive to stress may be more prone to sleep disturbance and that their psychological resilience should be adequately assessed and monitored. Individuals with intermediate and early chronotypes and those less affected by stress may significantly benefit from strategies aimed at enhancing resilience during their clinical placement. Furthermore, for students identified with low resilience, implementing stress management interventions will be crucial to alleviating the influence of sleep reactivity on sleep quality, irrespective of chronotype.
